# The Polymorphisms of Genes Encoding Catalytic Antioxidant Proteins Modulate the Susceptibility and Progression of Testicular Germ Cell Tumor

**DOI:** 10.3390/cancers14041068

**Published:** 2022-02-20

**Authors:** Uros Bumbasirevic, Nebojsa Bojanic, Marija Pljesa-Ercegovac, Marko Zivkovic, Tatjana Djukic, Milica Zekovic, Bogomir Milojevic, Boris Kajmakovic, Aleksandar Janicic, Tatjana Simic, Vesna Coric

**Affiliations:** 1 Clinic of Urology, University Clinical Center of Serbia, 11000 Belgrade, Serbia; urosbu@gmail.com (U.B.); bojanicnebojsa@gmail.com (N.B.); markoziv91@gmail.com (M.Z.); em2bogomir@yahoo.com (B.M.); mockay@gmail.com (B.K.); aleksandarmjanicic@gmail.com (A.J.); 2 Faculty of Medicine, University of Belgrade, 11000 Belgrade, Serbia; m.pljesa.ercegovac@gmail.com (M.P.-E.); tatjana.djukic@med.bg.ac.rs (T.D.); 3 Institute of Medical and Clinical Biochemistry, Faculty of Medicine, University of Belgrade, 11000 Belgrade, Serbia; 4 Centre of Research Excellence in Nutrition and Metabolism, Institute for Medical Research, National Institute of Republic of Serbia, University of Belgrade, 11000 Belgrade, Serbia; zekovicmilica@gmail.com; 5 Department of Medical Sciences, Serbian Academy of Sciences and Arts, 11000 Belgrade, Serbia

**Keywords:** testicular GCT, oxidative stress, polymorphism, redox biomarkers

## Abstract

**Simple Summary:**

Testicular cancer is the most common malignancy in the population of young and reproductively active men. The risk factors for its occurrence are not fully elucidated. Undescended testicle remains the main risk factor; however, more precise molecular studies associate genetic variations with susceptibility to testicular tumor development and progression. In this study, we found that specific variations in genes encoding antioxidant defense proteins confer risks of testicular cancer development and progression and, therefore, helps to identify subjects at higher risk, as well as those requiring additional diagnostics and more intensive forms of treatment.

**Abstract:**

The simultaneous analysis of redox biomarkers and polymorphisms encoding for regulatory and catalytic antioxidant proteins was performed in order to evaluate their potential role in the development of testicular germ cell tumor (GCT), as well as the progression of the disease. *NRF2* (rs6721961), *GSTM3* (rs1332018), *SOD2* (rs4880) and *GPX3* (rs8177412) polymorphisms were assessed in 88 patients with testicular GCT (52 with seminoma) and 88 age-matched controls. The plasma levels of 8-hydroxy-2′-deoxyguanosine (8-OHdG), thiol groups and the plasma activity of glutathione peroxidase were measured. A significant association between variant GPX3*TC+CC genotype and risk of overall testicular GCT, as well as seminoma development, was found. Moreover, carriers of variant SOD2*TT genotype were at almost 3-fold increased risk of seminoma development. Interestingly, combined SOD2*TT/GPX3*TC+CC genotype conferred a 7-fold higher risk for testicular GCT development. Finally, variant GSTM3*AC+CC genotype was associated with a higher risk for the development of advanced diseased. The presence of assessed genetic variants was not associated with significantly higher levels of redox biomarkers in both testicular GCT patients, as well as in those diagnosed with seminoma. In conclusion, the polymorphic expression of certain antioxidant enzymes might affect susceptibility toward testicular GCT development, as well as the progression of the disease.

## 1. Introduction

Approximately 95% of malignant neoplasms occurring in the testis are of germ-cell origin. Although considered to be a rare malignancy in the general population, testicular cancers are the most common type of solid tumors among men aged 15 to 40 and a principal cancer-associated morbidity cause in this group [1]. Testicular germ cell tumors (GCTs) exhibiting a wide spectrum of histological patterns, pathogenetic features and clinical profiles are classified into two major entities: seminoma and non-seminoma [2,3]. A wide array of clinical determinants has been suspected of being associated with the etiopathology of testicular GCT, including components of testicular dysgenesis syndrome, encompassing cryptorchidism as the most common element [2,4]. Other hypothesized risk factors include individual genetic aberrations intensifying the susceptibility to testicular GCT, several maternal factors (dominantly referring to intrauterine exposures and surrogates), personal health-related and lifestyle characteristics (age, race, comorbidities, reproductive health issues, diet, physical activity, scrotal trauma, as well as occupational and miscellaneous exposures) and geographic and temporal determinants [5]. Regardless of the expanding comprehension of the risk factors for testicular cancer, the pathogenesis of this disease is not completely elucidated and further studies are warranted. The effect of genetic variations on susceptibility to testicular GCT has been the subject of several studies. Previous research has identified potential gene candidates, as well as their respective single nucleotide polymorphisms (SNPs) [6], that could meet the criteria for the BEST (Biomarkers, EndpointS, And other Tools, Food and Drug Administration) category of risk biomarkers [7]. The assessment of genetic polymorphisms known to affect the defensive antioxidant capacity of cells, as well as detoxification processes in patients with testicular GCT, may provide a valuable contribution to this field of research.

Numerous studies have shown that oxidative stress, as a condition in which the balance between prooxidants and antioxidants is tipped toward prooxidants, can play a significant role in the tumor onset and progression, representing the hallmark of carcinogenesis [8]. Indeed, the elevated oxidative burden may lead to a saturation of defense mechanisms and can mediate cellular transformation towards limitless proliferative potential, avoidance of programmed cell death and invasiveness [9]. However, the double-faced role of oxidative stress in tumor biology is well known [10]. Another hallmark of cancer cells is metabolic and functional adaptation causing resistance against high oxidative stress. Namely, in the advanced stage of the disease, the role of oxidative stress is two-fold—on the one hand, free radicals act as promoters of disease progression, and on the other hand, they mediate anticancer effects of chemo and/or radiotherapy [11]. In an attempt to oppose the effects of reactive oxygen species, cancer cells induce the expression of enzymes representing the immediate antioxidant defense (superoxide dismutase, SOD) or the first line of antioxidant defense, including glutathione peroxidase (GPX), glutathione S-transferase (GST) and catalase [10].

Genetic polymorphisms occur in both non-coding and coding regions of genes not only for antioxidant enzymes but for certain regulatory antioxidant proteins as well. Nuclear factor-erythroid-2-related factor 2 (NRF2) orchestrates basal and stress-induced transcription of key players in glutathione and thioredoxin antioxidant systems, as well as certain enzymes involved in phase I and phase II detoxification of exogenous and endogenous compounds [12]. Among all NRF2 single nucleotide polymorphisms (SNPs), the highest attention was given to rs6721961 (−617C/A), located in the promoter region, which seems to modulate its transcription activity [13]. Within a vast array of NRF2 targeted genes are those encoding for certain GSTs, known for their antioxidant and detoxifying catalytic roles, in addition to their negative regulation of protein kinases involved in cellular survival, proliferation and apoptosis as well, by the means of protein–protein interactions [14]. Glutathione S-transferase M3 is a member of a *Mu* cytosolic class of GSTs, predominantly in terms of cancer development [15]. Among GSTM3-related SNPs, special attention was paid towards rs1332018 (A-63C), which is located in a transcription factor-binding site and splicing site, with predicted regulatory potential affecting the enzymes’ expression [16]. As mentioned earlier, GSTs are recognized as a first-line member of antioxidant defense along with GPX. The GPX family numbers eight isozymes (GPX1–8), with GPX3 being known as an extracellular enzyme that catalyzes the detoxification of hydro- and soluble lipid hydroperoxides by reduced glutathione [17]. Polymorphism in gene encoding GPX3 (rs8177412) is a part of GPX3 promoter haplotype responsible for the downregulation of gene transcription, resulting in affected plasma GPX3 activity [18]. However, the reaction of radical dismutation is being considered as an immediate antioxidant defense, readily catalyzed by SOD isoenzymes. In particular, SOD2 scavenges the superoxide anion radical in mitochondria. Its transport to mitochondria is known to be reduced by 30–40% as a result of gene SNP (rs4880), which consists of nucleotide substitution (T, thymine → C, cytosine), causing an amino acid substitution of valine (Val) with alanine (Ala) [19,20]. Consequently, altered expression and activity of both regulatory and catalytic antioxidant proteins modify inter-individual variability of antioxidant capacity and, therefore, define a unique redox profile.

The results on determining redox biomarkers in patients with testicular GCT are rather limited or even inconsistent. Moreover, there is a scarcity of data on the association of polymorphisms encoding for regulatory and catalytic antioxidant proteins in susceptibility to testicular GCT development. Therefore, the present study was conducted with an objective to provide the comprehensive analysis of biochemical indicators of oxidative damage and aforementioned genetic determinants, in a simultaneous manner, in order to evaluate their potential role in the development of testicular GCT, as well as the progression of the disease. Particular emphasis was given to seminoma as the most frequent histological type of testicular GCT.

## 2. Materials and Methods

**Figure 1:**
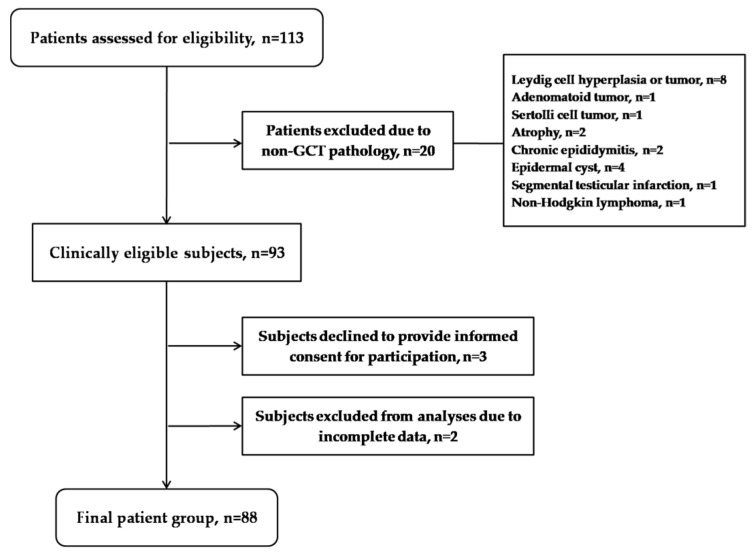
The flowchart detailing the patient enrollment process.

Patients with testicular masses were treated at the Clinic of Urology, University Clinical Centre of Serbia, between years 2020 and 2021. A total of 113 subjects were admitted during the studied period and assessed for eligibility. The inclusion criteria for this study comprised the following: age equal to or above 18 years, novel diagnosis of a testicular GCT (confirmed by physical and ultrasound examination, pathological assessment in accordance with the latest WHO classification [3,21,22]) and voluntary participation prior to treatment initiation. Exclusion criteria were as follows: the presence of other malignancies, ongoing cytotoxic or radiotherapy, compromising mental conditions, active psychiatric disorders and limited literacy/language skills. As indicated in Figure 1, twenty patients were excluded from the study due to non-GCT pathology, two as a result of incomplete data and three, although clinically eligible, refused to provide informed consent. Accordingly, the final group comprised 88 patients (average age 33.5 ± 8.7 years). Patients’ data were collected using the structured questionnaire (LymeSurvey based, https://upitnik.med.bg.ac.rs/, accessed on 4 January 2022) and clinical charts.

The control group was selected from DNA Biobank (formed at the Institute of medical and clinical biochemistry, Faculty of Medicine, University of Belgrade). Furthermore, the control group was properly age-matched and all malignant and non-malignant urological conditions that could possibly interfere with analyzed parameters were treated as exclusion criteria. Additionally, control subjects were selected from the same source population as the cases, thus limiting the confounding effect of ethnic background and geographical factors, eventually comprising 88 individuals (average age 35.1 ± 9.9 years). Informed written consent was obtained from all recruited subjects. The study was approved by the Institutional Ethical Board of Clinical Centre of Serbia, Serbia, and was performed in accordance with the principles of the Helsinki declaration.

DNA was isolated from blood collected in EDTA-coated tubes, using a commercial kit (Invitrogen™, PureLink™ Genomic DNA Mini Kit). The rest of the blood sample was used for obtaining plasma. NRF2 (rs6721961) polymorphism was determined by PCR-CTTP method [23], whereas *GSTM3* (rs1332018), *SOD2* (rs4880) and *GPX3* (rs8177412) polymorphisms were determined by qPCR using TaqMan™ Drug Metabolism Genotyping Assays (Applied Biosystems™, Thermo Fisher Scientific corporation, Waltham, MA, USA) numbered C_3184522_30, C_8709053_10 and C_25964717_20, respectively. The plasma level of 8-OHdG (8-Hydroxydeoxyguanosine) was measured by an 8-OHdG ELISA Kit (Elabscience, number E-EL-0028), and the concentration of thiol groups was measured spectrophotometrically, according to the method of Jocelyn [24,25]. The activity of plasma GPX was determined as described by Gunzler A. et al. [26].

Statistical data analysis was performed using IBM SPSS Statistics 22 (SPSS Inc., Chicago, IL, USA). In this study, categorical data variables were presented using frequency counts (*n*, %), whereas continuous data variables were in the majority of cases expressed as median (minimum–maximum). Apart from testing the differences between categorical variables, an χ2 test was used to assess whether the investigated genotypes were in the Hardy–Weinberg equilibrium. The differences in continuous data with non-normal distribution were assessed either by using Mann–Whitney or Kruskal–Wallis tests. The genetic variants and their risk for testicular GCT development and progression were computed by odds ratios (OR) and 95% confidence intervals (CI) by logistic regression analysis. The association of individual gene polymorphisms with the risk of testicular GCT development and progression was analyzed using two models: crude OR and OR adjusted to other genotypes.

## 3. Results

### 3.1. The Characteristics of Patients with Testicular GCT and Respective Controls

Selected characteristics of 88 testicular GCT patients and 88 controls are presented in Table 1. As shown, no statistical difference was found in terms of age, obesity and smoking (*p* < 0.05) between these groups. Moreover, the frequency of factors associated with higher risk for testicular GCT development was lower than 10%. The majority of patients were diagnosed with seminoma (59%), as well as clinical stage I (69%).

### 3.2. The Association of Polymorphisms Encoding for Regulatory and Catalytic Antioxidant Proteins with the Risk for Testicular GCT Development

The distributions of gene polymorphisms for regulatory and catalytic antioxidant proteins in patients with testicular GCT and the odds ratio of developing this type of tumor are shown in Table 2. The only significant difference was observed regarding the distribution of *GPX3* genotypes between patients and controls (*p* = 0.002, for χ2 test), as well as its association with the risk of testicular GCT development. Namely, it was determined that the carriers of variant GPX3*TC+CC genotype were at 2-fold increased risk of developing testicular GCT, compared to the carriers of the referent GPX3*TT genotype (Table 2: OR = 2.61, 95%CI: 1.40–4.86, *p* = 0.002), which was confirmed when adjusted (OR = 2.14, 95%CI: 1.09–4.19, *p* = 0.027). However, as the association of the variant SOD2*TT genotype with an increased risk of testicular GCT was near the threshold for statistical significance (Table 2: OR = 1.84, 95%CI: 0.91–3.71; *p* = 0.086; adjusted OR = 2.12, 95%CI: 0.97–4.62, *p* = 0.057 respectively), in the next step, we assessed the combined effect of these risk-related genotypes on susceptibility to testicular GCT.

As indicated in Table 3, the carriers of SOD2*TT/GPX3*TC + CC combined genotype exhibited a 7-fold increased risk for testicular GCT development in comparison with patients carrying the referent SOD2*CC + CT/GPX3*TT genotype combination (OR = 7.48, 95%CI: 2.26–24.70, *p* = 0.001).

Due to the homogenous pathological phenotype, we further focused on the group of seminoma patients included in this study (Table 4). In this subpopulation, the distributions of *GPX3* genotypes, as well as the conferred risk, were similar to the results obtained in the overall group of patients with testicular GCT (OR = 2.95, 95%CI: 1.44–6.06, *p* = 0.003; adjusted OR = 2.29, 95%CI: 1.04–5.06, *p* = 0.039). This time, a significant association between SOD2*TT genotype and the risk of seminoma development was observed (OR = 2.46, 95%CI: 1.12–5.41, *p* = 0.025; adjusted OR = 2.84, 95%CI: 1.20–6.74, *p* = 0.017), thus providing a more refined confirmatory analysis. Of note, the association of investigated polymorphisms with the risk of development of non-seminoma testicular GCT subtypes was not estimated, as the number of patients was insufficient for this type of statistical analysis.

### 3.3. The Association between Polymorphisms Encoding for Regulatory and Catalytic Antioxidant Proteins with the Risk of Disease Progression

The distributions of gene polymorphisms for regulatory and catalytic antioxidant proteins in patients with localized and advanced disease, as well as the risk for disease progression, are shown in Table 5. A statistically significant difference in *GSTM3* genotype distribution among patients who were diagnosed with clinical stage I compared to those who had higher clinical stages (II and III) was observed (*p* = 0.033 for χ2 test). Moreover, the carriers of the GSTM3*AC+CC genotype exhibited significantly higher risks of advanced disease development in comparison to the carriers of GSTM3*AA (OR = 2.95, 95%CI: 1.07–8.13, *p* = 0.036; adjusted OR = 4.51, 95CI%: 1.30–15.63, *p* = 0.018).

### 3.4. The Association between Polymorphisms Encoding for Regulatory and Catalytic Antioxidant Proteins with Redox Biomarkers in Patients with Testicular GCT

In the testicular GCT group of patients, the median values of 8-OHdG plasma concentration were 9.10 (4.29–49.50) ng/L, thiol group plasma concentration at 9.67 (4.98–28.86) µmol/g and the activity of plasma GPX 354.34 (179.42–591.00) U/L. In the group of seminoma patients, the median values of 8-OHdG plasma concentration were 9.21 (5.07–49.50) ng/L, thiol group plasma concentration at 9.56 (4.98–28.86) µmol/g and the activity of plasma GPX 363.66 (183.92–591.00) U/L. The plasma levels of these redox biomarkers in total patient testicular GCT sample, and in particular among seminoma patients, analyzed with respect to individual *NRF2, GSTM3, SOD2* and *GPX3* genotypes are provided in Supplementary Tables (Tables S1 and S2). Surprisingly, the obtained values did not indicate any statistical difference in the levels of measured redox biomarkers (*p* > 0.05).

## 4. Discussion

Reactive species may create an ambient for genetic lesions, tumorigenicity and subsequent cancer progression. Therefore, it is reasonable to assume that the presence of gene variants encoding for regulatory and catalytic antioxidant proteins, such as *NRF2, GTSM3, SOD2* and *GPX3* polymorphisms, functionally resulting in altered levels of antioxidant defense, may affect the risk of tumor development, including testicular tumors. By the same token, the question arises in terms of whether the polymorphism of the *NRF2, GSTM3, SOD2* and *GPX3* genes can affect the tumor progression, the prognosis of patients with testicular GCT and eventually the response to systemic chemotherapy. In this study, the GPX3*TC+CC genotype was significantly associated with the risk of developing testicular GCT, including the risk of developing seminoma. In addition, the carriers of the SOD2*TT genotype were at a higher risk of developing seminoma. Moreover, 22% of all recruited patients with testicular GCT were carriers of the combined SOD2*TT/GPX3*TC+CC genotype, which was associated with a significantly higher risk of developing testicular GCT. No impaired plasma levels of redox biomarkers were observed in patients with testicular GCT with respect to the analyzed genotypes. Nevertheless, the GSTM3*AC + CC genotype was associated with a higher risk of disease progression.

Disturbed redox homeostasis, which occurs either due to increased activity of the free radical production system, or due to insufficient antioxidant defense, is thought to affect the regulation of signaling pathways involved in cell proliferation, growth, survival and apoptosis and, therefore, cancer development [27]. The NRF2-Keap1-ARE (Nuclear factor-erythroid-2-related factor 2/Kelch-like ECH-associated protein 1/Antioxidant response element) stress–response pathway is involved in NRF2-mediated response to xenobiotic and oxidative stress, as it plays a fundamental role in maintaining the redox cellular homeostasis [12]. NRF2 -617C/A polymorphism (rs6721961) is positioned in the middle of the ARE motif, affecting the binding of NRF2 in the carriers of the NRF2*AA genotype [28], hence reducing the degree of mRNA expression for glutathione and thioredoxin antioxidant enzymes, as well as for enzymes involved in phase I and phase II detoxification of exogenous and endogenous products, NADPH regeneration and heme metabolism [12]. However, the results of this study indicated no association of *NRF2* polymorphism (rs6721961) with the risk of testicular GCT development, as well as with the risk of disease progression. Notably, the perception of this molecule in carcinogenesis has changed from an anticancer molecule towards a molecule supporting cancer cell survival, even being considered as a possible anticancer target molecule [29,30].

SOD2 catalyzes the dismutation of a superoxide, formed as a by-product of oxidative phosphorylation. Lower expressions of the SOD2 enzyme have been established in the cells of many tumors [19]. Several studies have demonstrated that the variant SOD2*TT (rs4880) genotype is associated not only with impaired mRNA stability and decreased enzyme synthesis but also with a lower levels of enzyme import into the mitochondrial matrix [20]. So far, previous research has indicated a significant impact of *SOD2* polymorphism (rs4880) on the increased risk for breast, lung, prostate, colon and ovarian cancer development [31]. The present study demonstrated the SOD2*TT genotype as a significant risk biomarker for seminoma development. Only a pilot research by Biggs et al. has shown a twice-increased risk of developing testicular tumors in carriers of the SOD2*CT, as well as carriers of the SOD2*TT genotype [32]. In addition, other studies have associated *SOD2* polymorphic expression with a significant risk for infertility, a recognized factor for testicular GCT development and with a higher degree of DNA fragmentation and 8-OHdG levels [33]. Nonetheless, altered plasma levels of not only 8-OHdG but other redox biomarkers determined in this study were not observed in testicular GCT carriers of *SOD2* variant genotypes. The results of this study also demonstrated that the variant GPX3*TC+CC was a significant risk factor for testicular GCT development and, in particular, seminoma development. Previous studies related the presence of the GPX3*C (rs8177412) variant allele with lower transcriptional activity and, thus, reduced intracellular expression of GPX3 enzyme in almost all examined tumor tissue samples, except in the case of ovarian cancer [17,34]. Supposedly, hypermethylation along with other alterations in the *GPX3* promoter region, such as SNPs, modify the enzyme expression and worsen the patients’ prognosis, most likely due to the regulatory role of GPX3 in certain signaling cascades [17,34]. However, in this study, the presence of variant GPX3*TC+CC genotype, known to decrease the expression of the GPX3 enzyme, was not associated with an increased risk of advanced disease. Since GPX3 is considered predominantly an extracellular isoenzyme, its activity is assumed to be additionally affected by the availability of cofactors necessary for its catalytic function, such as selenium and glutathione [35]. The results of this study did not show the decreased activity of plasma GPX in GPX3*TC+CC genotype carriers, both in the overall group of testicular GCT patients, as well as in those diagnosed with seminoma.

Interestingly, when risk associated genotypes were analyzed in combination, the obtained results showed that the SOD2*TT/GPX3*TC+CC genotype was associated with a significantly increased risk of testicular GCT. To the best of our knowledge, this is the first study to analyze the effect of both individual polymorphisms of catalytic antioxidant proteins, and their combined effect on the risk of developing testicular tumors. Hopefully, our findings may contribute to future focused Next Generation Sequencing (NGS) based research, aiming to assess the role of a novel panel of genes encoding antioxidant proteins in terms of testicular cancer susceptibility.

Glutathione transferases are well recognized for their dual roles. As catalytic proteins, they are traditionally recognized as phase II detoxification superfamily of enzymes [36] in addition to their antioxidant capabilities [37]. Still, the mentioned catalytic properties might be affected by non-synonymous SNPs that seem to exert deleterious effects, leading to carcinogenesis [38]. Indeed, several studies have shown that some polymorphisms occurring within the non-coding *GSTM3* gene region modulate the risk of developing certain tumors [15]. However, the herein assessed *GSTM3* (rs1332108) polymorphism did not prove to be a significant biomarker of risk for testicular GCT development, nor for its most common pathohistological subtype—seminoma. On the other hand, the variant GSTM3*AC+CC genotype was associated with a significantly higher risk of disease progression, given the fact that 72% of testicular GCT patients with advanced disease (clinical stage II and III) were carriers of GSTM3*C allele. This variant allele is found in the promotor gene region and is known to decrease the expression of the GSTM3 enzyme [16,39]. Indeed, Zimmermann et al. have demonstrated a decline in GSTM3 protein expression, analyzed in seminoma tumor tissue, depending on the stage of the disease [40]. In the case of clear cell renal cell carcinoma, the presence of variant GSTM3*C allele (rs1332108) was associated with lower GSTM3 protein expression in tumor tissue, in comparison with non-tumor tissue, as well as with poorer patients’ prognosis [16]. GSTM3 shares quite a degree of similarity with GSTM1 in terms of amino acid sequence [41] and linkage disequilibrium [42]. In addition, GSTM3 seems to exhibit non-catalytic roles similar to GSTM1, as a part of GST family dual repertoire [43,44], by being engaged in protein–protein interactions with signaling molecules involved in the negative regulation of apoptosis, such as TRAF6 (TNF receptor-associated factor 6) found in HeLa cells [45]. The possible molecular mechanism underlying the role of variant GSTM3*C allele in testicular GCT progression has yet to be deciphered.

Several limitations of this study should be acknowledged. Albeit the applied case-control study design is considered to be an appropriate and practical approach for the evaluation of risk factors for rare diseases such as testicular cancer, there is an inherent risk of selection bias that might compromise the validity of the obtained results. Unfortunately, the data on environmental or occupational exposure were not available; hence, they were not used in the analysis. The assessments of redox biomarker levels in patients’ tumor tissue and sperm samples, as well as plasma samples of control members, were outside the scope of this study but may be considered for future research perspectives. A relatively small sample size could explain the absence of significant results when the plasma biomarkers were analyzed concerning assessed polymorphisms and limit the generalizability of presented findings.

## 5. Conclusions

In conclusion, the polymorphic expression of certain antioxidant enzymes might affect susceptibility towards testicular GCT development. GSTM3 polymorphism might serve as a marker of disease progression and, therefore, contribute to better identification of patients requiring additional diagnostics and more intensive forms of treatment. Further studies, with a larger sample size is required to fully elucidate the changes in testicular GCT patients’ redox homeostasis, related to the polymorphisms occurring in the immediate and first line of enzymatic antioxidant defense.

## Figures and Tables

**Table 1:** The characteristics of patients with testicular GCT and selected controls.

Parameters	Testicular GCT Patients	Control Group	OR (95%CI)	*p*
Age (years)	33.5 (19–54) ^1^	36.5 (18–54) ^1^	-	0.266 ^2^
Body mass index, *n* (%) ^3^				
<30 kg/m^2^	73 (88)	63 (84)	1.00 ^4^	
>30 kg/m^2^	10 (12)	12 (16)	0.72 (0.29–1.77)	0.475 ^5^
Smoking status, *n* (%) ^3^				
Never	36 (44)	42 (48)	1.00 ^4^	
Ever	45 (56)	46 (52)	1.14 (0.62–2.09)	0.669 ^5^
Factors associated with higher risk for testicular GCT development, *n* (%) ^3^				
Cryptorchismus	8 (9)	-	-	-
Infertility	4 (5)	-	-	-
Family history	4 (5)	-	-	-
Testicular atrophy	7 (8)	-	-	-
Tumor type, *n* (%)				
Seminoma	52 (59)	-	-	-
Non-seminoma	36 (41)	-	-	-
Clinical stage, *n* (%)				
I	61 (69)	-	-	-
II	18 (21)	-	-	-
III	9 (10)	-	-	-

^1^ Median (Min–Max); ^2^ value for Student’s t-test; ^3^ data available; ^4^ reference group; ^5^ *p* value for logistic regression.

**Table 2:** The association of *NRF2, GSTM3, SOD2* and *GPX3* polymorphisms with the risk for testicular GCT development.

Genotype	Testicular GCT Patients, *n* (%)	Control Group, *n* (%)	OR (95%CI) ^1^	*p*	OR (95%CI) ^2^	*p*
*NRF2* (rs6721961)						
*AA*	2 (2)	3 (4)	1.00 ^3^		1.00 ^3^	
*CC + CA*	84 (98)	73 (96)	1.72 (0.28–10.61)	0.556	1.42 (0.20–9.72)	0.719
*GSTM3* (rs1332018)						
*CC*	10 (12)	19 (22)	1.00 ^3^		1.00 ^3^	
*AA + AC*	73 (88)	67 (78)	2.07 (0.90–4.76)	0.087	1.23 (0.48–3.10)	0.662
*SOD2* (rs4880)						
*CC + CT*	54 (67)	69 (79)	1.00 ^3^		1.00 ^3^	
*TT*	26 (33)	18 (21)	1.84 (0.91–3.71)	0.086	2.12 (0.97–4.62)	0.057
*GPX3* (rs8177412)						
*TT*	38 (44)	58 (67)	1.00 ^3^		1.00 ^3^	
*TC + CC*	48 (56)	28 (33)	2.61 (1.40–4.86)	0.002	2.14 (1.09–4.19)	0.027

^1^ OR—crude odds ratio; ^2^ OR adjusted to other genotypes; CI—confidence interval; ^3^ reference group.

**Table 3:** The combined effect of risk-related genotypes on the susceptibility to testicular GCT development.

Genotypes	Testicular GCT Patients, *n* (%) ^1^	Control Group, *n* (%) ^1^	OR (95%CI) ^2^	*p*
SOD2*CC + CT/GPX3*TT	25 (32)	44 (52)	1.00 ^3^	
SOD2*TT/GPX3*TC + CC	17 (22)	4 (5)	7.48 (2.26–24.70)	0.001

^1^ Percentage out of total number; ^2^ OR—crude odds ratio; CI—confidence interval; ^3^ reference group.

**Table 4:** The association of *NRF2, GSTM3, SOD2* and *GPX3* polymorphisms with the risk for seminoma development.

Genotype	Seminoma Patients, *n* (%)	Control Group, *n* (%)	OR (95%CI) ^1^	*p*	OR (95%CI) ^2^	*p*
*NRF2* (rs6721961)						
*AA*	2 (4)	3 (4)	1.00 ^3^		1.00 ^3^	
*CC + CA*	49 (96)	73 (96)	1.70 (0.16–6.24)	0.994	0.801 (0.32–2.70)	0.942
*GSTM3* (rs1332018)						
*CC*	7 (14)	19 (22)	1.00 ^3^		1.00 ^3^	
*AA + AC*	43 (86)	67 (78)	1.74 (0.67–4.49)	0.251	0.94 (0.32–2.70)	0.912
*SOD2* (rs4880)						
*CC + CT*	28 (61)	69 (79)	1.00 ^3^		1.00 ^3^	
*TT*	18 (39)	18 (21)	2.46 (1.12–5.41)	0.025	2.84 (1.20–6.74)	0.017
*GPX3* (rs8177412)						
*TT*	21 (41)	58 (67)	1.00 ^3^		1.00 ^3^	
*TC + CC*	30 (59)	28 (33)	2.95 (1.44–6.06)	0.003	2.29 (1.04–5.06)	0.039

^1^ OR—crude odds ratio; ^2^ OR adjusted to other genotypes; IP—confidence interval; ^3^ reference group.

**Table 5:** The association between polymorphisms encoding for regulatory and catalytic antioxidant proteins with the risk of disease progression.

Genotype	Stage I, *n* (%)	Stages II + III, *n* (%)	OR (95%CI) ^1^	*p*	OR (95%CI) ^2^	*p*
*NRF2* (rs6721961)						
*CC*	43 (72)	18 (69)	1.00 ^3^		1.00 ^3^	
*CA + AA*	17 (28)	8 (31)	1.12 (0.41–3.06)	0.819	0.82 (0.22–3.01)	0.769
*GSTM3* (rs1332018)						
*AA*	31 (53)	7 (28)	1.00		1.00 ^3^	
*AC + CC*	27 (47)	18 (72)	2.95 (1.07–8.13)	0.036	4.51 (1.30–15.63)	0.018
*SOD2* (rs4880)						
*CC*	11 (20)	7 (28)	1.00 ^3^		1.00 ^3^	
*CT + TT*	44 (80)	18 (72)	0.64 (0.21–1.92)	0.429	0.389 (0.14–1.45)	0.160
*GPX3* (rs8177412)						
*TT*	28 (47)	10 (38)	1.00 ^3^		1.00 ^3^	
*TC + CC*	32 (53)	16 (62)	1.40 (0.54–3.58)	0.482	2.21 (0.69–7.09)	0.182

^1^ OR—crude odds ratio; ^2^ OR adjusted to other genotypes; IP—confidence interval; ^3^ reference group.

## Data Availability

The data supporting reported results can be found upon request in the form of datasets available at Clinic of Urology, University Clinical Centre of Serbia and LymeSurvey repository, https://upitnik.med.bg.ac.rs/, accessed on 4 January 2022, Institute of Medical and Clinical Biochemistry, Faculty of Medicine, University of Belgrade.

## Supplementary Materials

The following supporting information can be downloaded at: https://www.mdpi.com/article/10.3390/cancers14041068/s1. Table S1. Levels of plasma redox biomarkers in testicular GCT patients, carriers of individual *NRF2, GSTM3, SOD2* and *GPX3* genotypes; Table S2. Levels of plasma redox biomarkers in seminoma patients, carriers of individual *NRF2, GSTM3, SOD2* and *GPX3* genotypes.
