# Supplementary material for: The Polymorphisms of Genes Encoding Catalytic Antioxidant Proteins Modulate the Susceptibility and Progression of Testicular Germ Cell Tumor
Source: Cancers (Basel). 2022 Feb 20;14(4):1068. doi: 10.3390/cancers14041068 (PMC8870690; doi:10.3390/cancers14041068)
Supplement: Supplementary file 1 [file cancers-14-01068-s001.zip › cancers-1566275-supplementary.pdf]

Table S1. Levels of plasma redox biomarkers in testicular GCT patients, carriers of individual *NRF2, GSTM3, SOD2* and *GPX3* genotypes.

Genotypes	8-OHdG (ng/mL)[1]	*p*	Thiol Group (μmol/g)[1]	*p*	GPX Activity (U/L)[1]	*p*
			NRF2 (rs6721961)			
AA	-	-	10.00 (8.62–11.39)	-	476.52 (445.98–507.07)	-
CC+CA	-	-	9.78 (4.98–28.86)	0.744	353.37 (1.12–8.69)	0.036
			GSTM3 (rs1332018)			
CC	8.75 (4.29–11.83)	-	8.71 (5.93–13.35)	-	352.89 (245.02–564.31)	-
AA+AC	9.27 (5.07–49.50)	0.349	9.67 (4.98–14.89)	0.395	357.39 (179.42–591.00)	0.585
			SOD2 (rs4880)			
CC+CT	9.01 (4.29–49.50)	-	9.92 (4.98–28.86)	-	358.52 (179.42–591.00)	-
TT	9.54 (6.54–16.48)	0.725	9.61 (7.89–12.15)	0.613	337.62 (189.39–580.39)	0.979
			GPX3 (rs8177412)			
TT	10.64 (5.07–49.50)	-	9.92 (6.46–28.86)	-	359.80 (265.92–591.00)	-
TC+CC	9.02 (4.29–31.49)	0.947	9.63 (4.98–15.92)	0.226	353.37 (179.42–580.39)	0.380

[1]Median (Min-Max).

Table S2. Levels of plasma redox biomarkers in seminoma patients, carriers of individual *NRF2, GSTM3, SOD2,* and *GPX3* genotypes.

Genotypes	8-OHdG (ng/mL)[1]	*p*	Thiol Group (μmol/g)[1]	*p*	GPX Activity (U/L)[1]	*p*
			NRF2 (rs6721961)			
AA	-	-	10.00 (8.62–11.39)	-	476.52 (445.98–507.07)	-
CA+AA	-	-	9.63 (4.98–28.86)	0.686	359.96 (183.92–591.00)	0.049
			GSTM3 (rs1332018)			
CC	8.99 (8.48–11.83)	-	8.71 (6.62–13.35)	-	336.97 (265.92–564.31)	-
AA+AC	9.25 (5.07–49.50)	0.858	9.62 (4.98–11.70)	0.922	363.98 (183.92–591.00)	0.585
			SOD2 (rs4880)			
CC+CT	9.25 (5.07–49.50)	-	9.56 (4.98–28.86)	-	363.66 (183.92–591.00)	-
TT	9.03 (6.54–15.10)	0.564	9.67 (7.96–12.15)	0.362	346.30 (189.39–580.39)	0.961
			GPX3 (rs8177412)			
TT	9.25 (5.07–49.50)	-	10.00 (6.62–28.86)	-	363.66 (265.92–591.00)	-
TC+CC	9.03 (6.27–32.13)	0.585	9.62 (4.98–12.15)	0.280	364.30 (183.92–580.39)	0.944

[1]Median (Min-Max).
